# Effects of Combined Anisodamine and Neostigmine Treatment on the Inflammatory Response and Liver Regeneration of Obstructive Jaundice Rats after Hepatectomy

**DOI:** 10.1155/2014/362024

**Published:** 2014-11-12

**Authors:** Chong-Hui Li, Xuan Zhang, Xin-Lan Ge, Xin Huang, Ai-Qun Zhang, Wan-Qing Gu

**Affiliations:** Department & Institute of Hepatobiliary Surgery, Chinese PLA General Hospital, Chinese PLA Medical College, Beijing 100853, China

## Abstract

*Background*. Cholestasis is associated with high rates of morbidity and mortality in patients undergoing major liver resection. This study aimed to evaluate the effects of a combined anisodamine and neostigmine (Ani+Neo) treatment on the inflammatory response and liver regeneration in rats with obstructive jaundice (OJ) after partial hepatectomy. *Materials and Methods*. OJ was induced in the rats by bile duct ligation. After 7 days biliary drainage and partial hepatectomy were performed. These rats were assigned to a saline group or an Ani+Neo treatment group. The expressions of inflammatory mediators, liver regeneration, and liver damage were assessed at 48 h after hepatectomy. *Results*. The mRNA levels of TNF-*α*, IL-1*β*, IL-6, MCP-1, and MIP-1*α*, in the remnant livers, and the serum levels of TNF-*α* and IL-1*β* were substantially reduced in the Ani+Neo group compared with saline group (*P* < 0.05). The Ani+Neo treatment obviously promoted liver regeneration as indicated by the liver weights and Ki-67 labeling index (*P* < 0.05). The serum albumin and *γ*-GT levels and liver neutrophil infiltration also significantly improved in the Ani+Neo group (*P* < 0.05) compared with the saline group. *Conclusions*. These results demonstrate that the combined anisodamine and neostigmine treatment is able to improve the liver regeneration in rats with OJ by substantially alleviating the inflammatory response.

## 1. Introduction

In the presence of cholestasis, liver surgery is associated with high incidences of postoperative complications and even death [1, 2]. Hepatic inflammation is a significant feature of cholestatic liver disease. Proinflammatory cytokines, such as tumor necrosis factor-*α* (TNF-*α*), interleukin-6 (IL-6), and interleukin-1*β* (IL-1*β*), play important roles in the pathological damage caused by obstructive jaundice (OJ) and are the principal molecules involved in the high incidence of perioperative complications and high mortality rates associated with cholestatic liver disease [3–5]. The development of new methods aimed at reducing the inflammatory response and its effects on the cholestatic liver may improve the safety of liver surgery.

In experimental models of inflammatory diseases, vagus nerve stimulation attenuates the production of proinflammatory cytokines and inhibits the inflammatory process [6]. At the molecular level, most studies on the anti-inflammatory potential of the vagus nerve have been based on the effects of acetylcholine, which is the principal neurotransmitter of the parasympathetic nervous system. Acetylcholine interacts with the *α*7-nicotinic acetylcholine receptor (*α*7nAChR) that is expressed in macrophages and other cells and inhibits the production of proinflammatory cytokines to attenuate the local and systemic inflammatory responses [7, 8].

Anisodamine, which is a muscarinic acetylcholine receptor antagonist, has been used for clinical treatment of various types of shock in China since 1965. Recent studies have linked its antishock effects to the *α*7nAChR-dependent anti-inflammatory pathway and have demonstrated that anisodamine is able to indirectly increase the binding of endogenous acetylcholine to *α*7nAChR [9]. Neostigmine, which is an agent frequently used to reverse the effects of muscle relaxants during surgery, also exerts profound cholinomimetic effects due to its inhibition of the peripheral anticholinesterase enzyme [10, 11]. A combined anisodamine and neostigmine treatment has been proven to augment the antishock efficacy in a murine endotoxic shock model and a dog hemorrhagic shock model [12] and to inhibit joint inflammation in collagen-induced arthritis mice [13]. Notably, anisodamine not only increases the binding of ACh to *α*7nAChR but also blocks the side effects induced by neostigmine via muscarinic acetylcholine receptors.

This study was designed to evaluate the therapeutic effects of anisodamine combined with neostigmine on the inflammatory response and liver regeneration in bile duct ligation (BDL) induced OJ rats after partial hepatectomy.

## 2. Materials and Methods

### 2.1. Animals

Male Wistar rats weighing 240–260 g were provided by the Experimental Animal Center of the Academy of Military Medical Science (Beijing, China). The rats were maintained at 24°C under pathogen-free conditions under a 12 : 12-h dark/light cycle and were allowed food and water* ad libitum*. The procedures in this experiment were approved by the Animal Ethics Committee of Chinese PLA General Hospital.

### 2.2. Induction of OJ

The rats were fasted but had free access to water for 12 h before the surgery. The surgical procedures were performed under ether anesthesia using a surgical operating microscope. To establish OJ, a laparotomy was performed with a median incision. The common bile duct was isolated and resected after double ligations of the proximal and distal bile duct with 5-0 silk to prevent recanalization of the duct. Then, the abdomen was closed.

### 2.3. Biliary Drainage and Hepatectomy

Seven days after bile duct ligation, a second laparotomy was performed under ether anesthesia. Biliary drainage was restored in situ by reconnecting the proximal end and the distal end of the common bile duct by inserting an 8-mm long stent prepared from a sterile intravascular catheter (Shanghai Pudong Jinhuan Medical Co., Ltd., Shanghai, China). The stent was tied in position using a purse-string suture. Then, a 68% hepatectomy was performed according to Higgins and Anderson [14]. The resected liver lobes were weighed to calculate the total liver weight.

### 2.4. Medical Treatment and Sample Collection

After biliary drainage and hepatectomy, the rats were randomly divided into the following two groups: a combined anisodamine and neostigmine treatment group (Ani+Neo) and a saline control group (*n* = 6 per group). Anisodamine (Shanghai Xinyijinzhu Pharmaceutical Co., Ltd., Shanghai, China) at 25 mg/kg and neostigmine (Tianjin Jinyao Amino Acid Co., Ltd., Tianjin, China) at 50 *μ*g/kg were administered by intraperitoneal injection. The treatment was initiated immediately after the hepatectomy and was continued twice per day for 2 days. The rats in the saline control group were injected with saline, and an additional 6 rats were assigned to the sham operation group.

At 48 h after the 2nd operation, blood samples were collected from the inferior vena cava for the biochemical tests and inflammatory factor measurements. The regenerated liver lobes were weighed and sliced into two portions using a razorblade. One portion was immediately frozen in liquid nitrogen and stored at −80°C for the determination of gene expression. The other portion was further sliced and fixed with 10% formaldehyde in 0.01 M phosphate buffer (pH 7.4) for the histopathological analysis.

### 2.5. Measurement of Indocyanine Green (ICG) Plasma Disappearance Rate (PDR)

The ICG for injection (Dandong Medical Co., Ltd., Liaoning, China) was dissolved in sterile water to a final concentration of 2.5 mg/mL. The inferior caval vein was used for the blood samplings performed at 1, 3, and 5 min after the freshly prepared ICG was injected into the femoral vein (2.5 mg/kg). The plasma samples were diluted (50 *μ*L plasma in 950 *μ*L 1% wt/vol bovine serum albumin and 0.9% wt/vol NaCl in water) and measured spectrophotometrically at 805 nm. The PDR was derived from the slope of the semilogarithmic decay curve [15].

### 2.6. Blood Biochemical Tests

To assess the damage to the hepatic parenchyma, the levels of serum alanine aminotransferase (ALT), aspartate aminotransferase (AST), albumin (ALB), total bilirubin (T-Bil), and *γ*-glutamyl transferase (*γ*-GT) were measured using standard laboratory methods and a serum analyzer (Cobas-Mira Plus, Roche Diagnostics, Manheim, Germany).

### 2.7. Assessment of Hepatic Regeneration

Two independent markers of liver regeneration assessment were used as follows: the regeneration of the liver mass was expressed as the percentage of the remnant liver weight at sacrifice relative to the estimated total liver weight at the time of partial hepatectomy and was calculated using the following equation: liver regeneration (%) = *A*/*B* × 0.68 × 100, where *A* represents the regenerated remnant liver weight at sacrifice and *B* represents the resected liver weight accounting for 68% of the entire liver at the time of hepatectomy. To assess hepatocyte proliferation, the Ki-67 labeling index was determined by Ki-67 immunostaining as follows: the fixed liver tissues were embedded, sectioned, and immunostained with a mouse anti-Ki-67 monoclonal antibody (BD Pharmingen) according to the manufacturer's instructions. The immunostained tissue was lightly counterstained with hematoxylin. The Ki-67 labeling index was determined as the percentage of Ki-67-positive hepatocytes per total number of hepatocytes in 10 random visual fields (400x). The histological analyses were performed in a blinded fashion with respect to the experimental groups.

### 2.8. Histopathological Analysis

The liver specimens were embedded in paraffin, cut into 5-*μ*m sections, and stained with hematoxylin and eosin. The hepatic neutrophil infiltration was detected by myeloperoxidase (MPO) immunostaining with a rabbit antibody (Pierce Biotechnology, Rockford, USA). A light microscope was used to verify the histopathological findings.

### 2.9. Determination of Hepatic mRNA Expression

The total RNA was extracted from the frozen liver tissues using the RNASimple Total RNA Kit (Tiangen Biotech, Beijing, China) according to the manufacturer's protocol; 4 *μ*g of RNA was reverse-transcribed with the RevertAid First Strand cDNA Synthesis Kit using oligo-dT primers (Thermo Scientific). Quantitative real-time polymerase chain reaction (qPCR) amplifications were performed using the SYBR Premix Ex Taq II (TakaRa) with the StepOnePlus Real Time PCR System (Applied Biosystems). The primers are shown in Table 1. The relative mRNA expression levels were normalized to the endogenous reference gene, glyceraldehyde-3-phosphate dehydrogenase (GAPDH), and calculated using the 2-ΔΔ Ct method as previously described [16]. The mRNA levels of the experimental groups were expressed as fold changes versus the sham operation group.

### 2.10. ELISA Assay of Serum Cytokine Levels

The serum levels of TNF-*α*, IL-6, and IL-1*β* were determined using commercial enzyme-linked immunosorbent assay (ELISA) kits (Quantikine Immunoassay, R&D system, Minneapolis, USA) according to the manufacturer's protocol using the serum samples.

### 2.11. Statistical Analysis

The results were expressed as the mean ± SD. Student's *t*-test was used to compare the two groups. The comparisons among more than 2 groups were performed by one-way analysis of variance (ANOVA) with post hoc multiple comparisons using SPSS version 17.0 (SPSS, Inc., Chicago, IL). Values of *P* < 0.05 were considered statistically significant.

## 3. Results

### 3.1. Anisodamine/Neostigmine Treatment Inhibited the Expression of Inflammatory Factors

**Figure 1:**
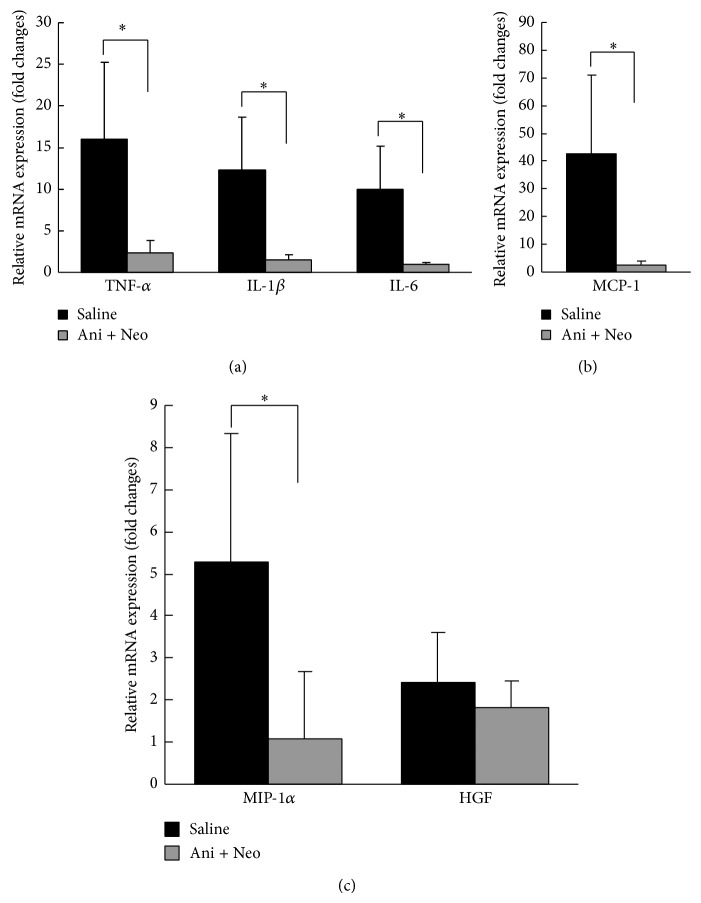
Quantitative reverse transcription PCR detection of the mRNA levels of inflammatory cytokines (TNF-*α*, IL-1*β*, and IL-6), chemokines (MCP-1 and MIP-1), and hepatocyte growth factor (HGF) in the cholestatic remnant livers at 48 h after biliary drainage and hepatectomy following treatment with anisodamine/neostigmine (Ani+Neo) or saline (saline). *n* = 6 for each group. The data are expressed as the mean ± SD relative to the values obtained in the sham-operated rats, which were arbitrarily assigned values of 1. _ _^*^ *P* < 0.05.

The local and systemic inflammatory responses were assessed by detecting the mRNA levels of the inflammatory factors in the regenerated livers and the serum levels of the inflammatory cytokines. The mRNA levels of the inflammatory cytokines (TNF-*α*, IL-1*β*, and IL-6), the chemokines [monocyte chemotactic protein 1 (MCP-1) and macrophage inflammatory protein-1 *α* (MIP-1*α*)], and the hepatocyte growth factor (HGF) in the cholestatic remnant liver were measured using reverse transcription qPCR. As shown in Figure 1, substantially reduced gene expression levels of TNF-*α*, IL-1*β*, IL-6, MCP-1, and MIP-1*α* were observed in the Ani+Neo group compared with the saline group (*P* < 0.05) at 48 h after the partial hepatectomy. No difference was observed in HGF expression between the saline and the Ani+Neo groups.

**Figure 2:**
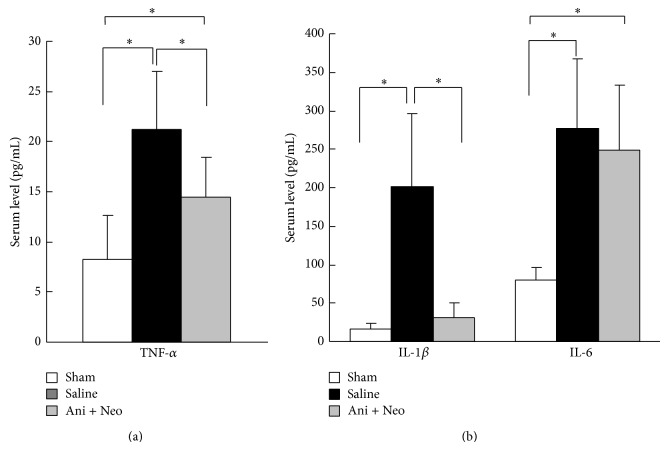
ELISA quantifications of the serum levels of TNF-*α*, IL-1*β*, and IL-6 in the sham operation group, the saline control group, and the combined anisodamine and neostigmine treatment (Ani+Neo) group (*n* = 6 for each group). _ _^*^ *P* < 0.05.

The serum levels of TNF-*α*, IL-1*β*, and IL-6, which were representative of proinflammatory cytokines, were detected using ELISA (Figure 2). The results showed that the Ani+Neo group had significantly lower serum levels of TNF-*α* and IL-1*β* than the saline group (*P* < 0.05). There was no difference in the IL-6 serum levels between the saline and the Ani+Neo groups, although these levels were higher than that in the sham group (*P* < 0.05).

### 3.2. Anisodamine/Neostigmine Treatment Improved Liver Regeneration

**Figure 3:**
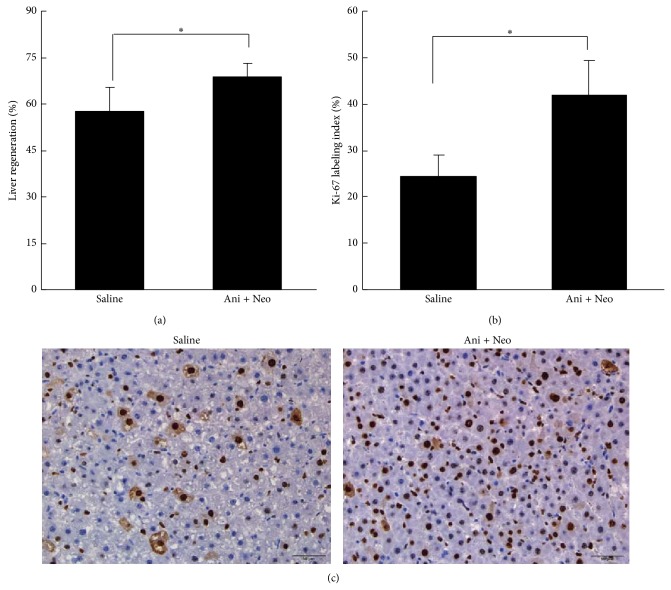
Liver regeneration was assessed by determining the percentage of remnant liver weight relative to the initial total liver weight (a) at 48 h after partial hepatectomy and the Ki-67 labeling index (b) with the representative histology (c) in the Ani+Neo group and the saline control group. _ _^*^ *P* < 0.05.

The regeneration of the remnant liver at 48 h after biliary drainage and partial hepatectomy was evaluated. The remnant livers of the saline and Ani+Neo group rats regenerated to 57.7 ± 7.8% and 68.9 ± 4.3% of their initial total liver weights, respectively (Figure 3(a)). Hepatic regeneration in the Ani+Neo group was significantly faster than that in the saline group (*P* < 0.05). The proliferation of hepatocytes, as indicated by the Ki-67 labeling index, was higher in the Ani+Neo group than in the saline group (*P* < 0.05) (Figures 3(b) and 3(c)). These results demonstrated that the combined anisodamine/neostigmine treatment improved liver regeneration in the cholestatic rats after hepatectomy.

### 3.3. ICG PDR in Regenerated Liver

**Figure 4:**
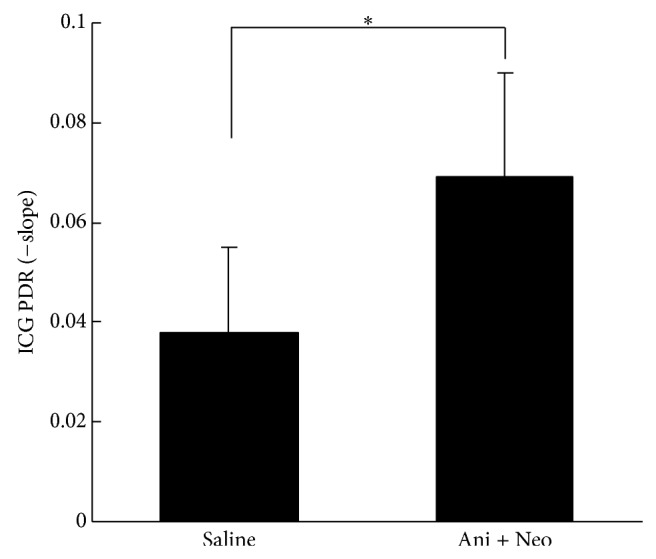
Indocyanine green (ICG) plasma disappearance rate (PDR) of the Ani+Neo group and the saline control group at 48 h after hepatectomy. _ _^*^ *P* < 0.05.

As a marker for the maximum elimination capacity of the liver, the indocyanine green PDR was assessed to determine the liver functioning of the cholestatic livers after partial hepatectomy. As illustrated in Figure 4, the PDR of the Ani+Neo group was significantly higher than that of the saline group at 48 h after hepatectomy (*P* < 0.05), suggesting the improved recovery of the elimination capacity of the hepatocytes and the biliary system.

### 3.4. Influence of Anisodamine/Neostigmine Treatment on Liver Damage

At 48 h after biliary drainage and hepatectomy, the OJ rats still showed elevated serum ALT, AST, T-Bil, and *γ*-GT levels and decreased albumin levels in the saline and Ani+Neo groups compared with those in the sham group (*P* < 0.05), indicating that hepatocytic and biliary injury were present (Table 2). The ALB of the Ani+Neo group was significantly higher than the corresponding value in the saline group, and the *γ*-GT level of the Ani+Neo group was much lower than that in the saline group, indicating changes consistent with liver regeneration.

**Figure 5:**
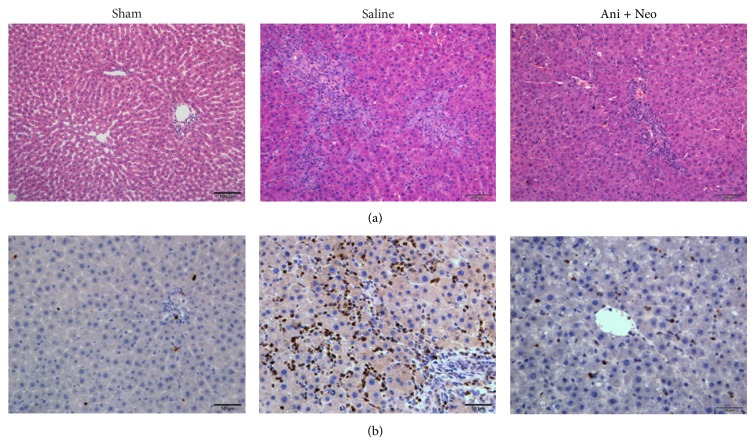
The histopathological changes in the rat livers from the sham operation, saline, and combined anisodamine/neostigmine treatment (Ani+Neo) groups at 48 h after hepatectomy based on hematoxylin and eosin staining ((a), 200x) and myeloperoxidase immunostaining ((b), 400x).

Additionally, histopathological liver alterations in the saline and Ani+Neo group rats were observed and compared with those in the sham group. Histopathological analysis of the liver tissues from the sham-operated rats revealed normal liver lobule structures with clear and complete hepatic cords. There was no bile duct hyperplasia or hepatocytic injury observed (Figure 5(a)). In the rat livers from the saline and Ani+Neo groups, cholestasis-induced bile duct hyperplasia was still obvious at 48 h after biliary decompression and hepatectomy, and there was no significant difference between the two groups with regard to the degree of bile duct hyperplasia (Figure 5(a)). Although swelling and vacuolar hepatocytes were noticeable in the saline and Ani+Neo groups, there were less vacuolar hepatocytes and more regenerated hepatocytes in the Ani+Neo group than in the saline group. The hepatic neutrophil influx as assessed by MPO immunostaining was more apparent in the saline group than in the Ani+Neo group (Figure 5(b)). These results suggested that the systemic application of the Ani+Neo treatment reduced the cholestasis-induced migration and infiltration of neutrophils and the hepatocyte injury.

## 4. Discussion

In this study, the BDL-induced OJ rats were administered treatments of combined anisodamine and neostigmine after 70% hepatectomy. The results demonstrated that the combined anisodamine and neostigmine treatment was effective in inhibiting the cholestasis-induced inflammatory response and improving liver regeneration in the cholestatic rats.

Anisodamine (6-[s]hydroxyhyoscyamine) is a naturally occurring tropane alkaloid found in plants in the Solanaceae family [17]. It is an anticholinergic drug that does not cross into the central nervous system. Anisodamine has been more widely used clinically in China for the treatment of various types of shock (particularly septic shock) because it has less severe adverse effects compared with atropine [18, 19]. Sun et al. [12] first demonstrated that the combined use of anisodamine and neostigmine is more effective than either drug alone for controlling inflammation. In the *α*7nAChR-knockout mice, this combined treatment does not improve the survival rate of septic mice, suggesting that the beneficial effects of combining anisodamine/neostigmine on endotoxic shock are mediated primarily by the *α*7nAChR.

In our experiments, combined anisodamine and neostigmine administration to the cholestatic rats that underwent partial hepatectomy was able to consistently decrease the mRNA levels of the inflammatory cytokines TNF-*α*, IL-1*β*, and IL-6 and the chemokines, MCP-1 and MIP-1*α*, in the remnant livers; however, this treatment had no effect on the expression of the HGF. MCP-1 and MIP-1*α* are important members of the chemokine family that are primarily involved in attracting neutrophils and monocytes to sites of inflammation [20, 21]. The serum levels of TNF-*α* and IL-1*β* were also significantly attenuated in the Ani+Neo group.

The regenerative capacity of the liver is a significant factor following liver surgery. Biliary obstruction has been shown to be an important pathological condition that inhibits hepatic regeneration [22]. Cholestasis leads to increased risks of accumulation of potentially toxic bile acids, chronic Kupffer cell activation, and deterioration of nutritional statuses [23]. During the early phases following BDL, Kupffer cells are activated as a result of significant portal endotoxemia, which results in increased phagocytic activity, production of cytokines, and release of toxic radicals and proteases [24]. Additionally, activated Kupffer cells release neutrophil-attracting chemokines, resulting in the accumulation of systemic activated neutrophils in the liver following BDL [3, 25]. Levy et al. [26] reported enhanced neutrophil activities within 12 h of bile duct ligation, and the increased activity levels remained for 15 days. Activated neutrophils release reactive oxygen intermediates, and these toxic substances are capable of damaging the sinusoidal endothelial cells and hepatocytes directly or by inducing other inflammatory mediators. In addition, IL-1*β* is a known inhibitor of liver regeneration and a proinflammatory cytokine that induces the production of other proinflammatory cytokines, particularly TNF-*α* and IL-6 [27]. Thus, activated Kupffer cells and infiltrated neutrophils contributed to the inflammation and impaired liver regeneration observed during OJ.

Our results showed that the combined administration of anisodamine and neostigmine ameliorated inflammatory cytokine and chemokine levels, resulting in reduced neutrophil infiltration in the remnant livers. This reduction in neutrophil infiltration may have contributed to the improvement of liver regeneration in the cholestatic hepatectomized rats. HGF is a cytokine that plays a crucial role in tissue regeneration by stimulating cell growth, cell motility, and morphogenesis [28]. The combined anisodamine and neostigmine treatment did not influence the expression of HGF in the remnant liver. This finding indicated the specific role of anisodamine and neostigmine in the cholinergic anti-inflammatory pathway.

ICG is a fluorescent agent that is eliminated exclusively by the liver via an energy-dependent transport mechanism when injected into a vein and its clearance from the blood depends on liver microcirculation, hepatocyte function, and biliary excretion [29]. The ICG plasma disappearance rate has become an important parameter that is measured before hepatectomy, and it is associated with morbidity and mortality after liver resection [30]. Consistent with the improved liver regeneration, as shown by the liver weights and the Ki-67 labeling index, the results of the ICG PDR demonstrated faster rates of liver regeneration and hepatocellular and biliary functional recoveries of the cholestatic livers following biliary drainage and partial hepatectomy.

The bile duct ligation led to complete OJ, and the serum ALT, AST, T-Bil, and *γ*-GT levels increased at the time of the hepatectomy [31]. In our study, biliary drainage was performed before the hepatectomy to mimic clinical situations. This procedure led to marked decreases in the serum ALT, AST, T-Bil, and *γ*-GT levels compared with the levels observed in rats with OJ (data not shown). There were no significant differences in the serum ALT, AST, and T-Bil levels between the Ani+Neo and saline groups. Following the biliary drainage and the improvements in liver regeneration, the ALB level in the Ani+Neo group increased and the *γ*-GT level decreased compared with those in the saline group. Although the histopathological changes in bile duct hyperplasia were comparable between the two groups following biliary drainage and hepatectomy, the significantly reduced MPO-positive cells and injured hepatocytes suggested that the mechanism underlying the combined anisodamine and neostigmine treatment may have involved reduction in the inflammatory response and neutrophil infiltration.

Both anisodamine and neostigmine have been used in clinical practice for many years; therefore, they are considered to be safe, although they are not typically used to treat inflammatory diseases. Because of their effects on the cholinergic pathway, the anti-inflammatory properties of both anisodamine and neostigmine were recently investigated in septic shock animal models. Neostigmine administered at a dose of 0.1 mg/kg is not protective against histopathologic organ injury due to endotoxemia, and a higher dose is not tolerated, which is likely because of nonspecific parasympathetic activities, including those involving the cardiovascular system [32]. The combination of anisodamine and neostigmine is able to not only enhance anti-inflammatory effects by blocking muscarinic receptors and thus mobilize ACh to bind to the *α*7nAChR but also abolish the side effects caused by muscarinic receptor-mediated vagus nerve activation. However, much work needs to be performed to successfully apply the anisodamine/neostigmine cocktail in clinical practice.

## 5. Conclusions

The combination of anisodamine and neostigmine substantially decreases the OJ-related inflammatory response, reduces liver injury, and improves remnant liver regeneration and functional recovery in rats with OJ after partial hepatectomy. This method of combined anisodamine and neostigmine treatment may be clinically useful for patients with cholestasis and hepatectomy.

## Figures and Tables

**Table 1:** Primer sequences.

Genes	Forward (5′-3′)	Reverse (5′-3′)
GAPDH	ACCACAGTCCATGCCATCAC	TCCACCACCCTGTTGCTGTA
TNF-*α*	AAATGGGCTCCCTCTCATCAGTTC	TCTGCTTGGTGGTTTGCTACGAC
IL-1	CACCTCTCAAGCAGAGCACAG	GGGTTCCATGGTGAAGTCAAC
IL-6	ACAGCGATGATGCACTGTCAG	ATGGTCTTGGTCCTTAGCCAC
HGF	TCCTGTGCCAAAACAAAACA	GGTGCTGACTGCATTTCTCA
MCP-1	AGCATCCACGTGCTGTCTC	GATCATCTTGCCAGTGAATGAG
MIP-1*α*	GCGCTCTGGAACGAAGTCT	GAATTTGCCGTCCATAGGAG

**Table 2:** The serum biochemical markers of liver injury and cholestasis.

Groups	ALT (U/L)	AST (U/L)	ALB (g/L)	T-Bil (mol/L)	*γ*-GT (U/L)
Sham	39.6 ± 5.7	117.6 ± 35.0	40.4 ± 1.8	0.4 ± 0.5	0.7 ± 0.4
Saline	89.3 ± 34.2^*^	421.9 ± 176.1^*^	27.2 ± 3.9^*^	25.6 ± 14.4^*^	13.9 ± 5.6^*^
Ani + Neo	79.4 ± 23.0^*^	352.5 ± 133.7^*^	32.2 ± 3.4^∗#^	19.7 ± 8.1^*^	6.4 ± 2.1^∗#^

Note: ^*^versus sham group, *P* < 0.05; ^#^versus saline group, *P* < 0.05.

## References

[1] Suda K., Ohtsuka M., Ambiru S., Kimura F., Shimizu H., Yoshidome H., Miyazaki M. (2009). Risk factors of liver dysfunction after extended hepatic resection in biliary tract malignancies. *The American Journal of Surgery*.

[2] Nanashima A., Abo T., Arai J., Matsumoto H., Kudo T., Nagayasu T. (2013). Functional liver reserve parameters predictive for posthepatectomy complications. *Journal of Surgical Research*.

[3] Saito J. M., Maher J. J. (2000). Bile duct ligation in rats induces biliary expression of cytokine- induced neutrophil chemoattractant. *Gastroenterology*.

[4] Gujral J. S., Farhood A., Bajt M. L., Jaeschke H. (2003). Neutrophils aggravate acute liver injury during obstructive cholestasis in bile duct-ligated mice. *Hepatology*.

[5] Aller M. A., Arias J. L., Prieto I., Losada M., Arias J. (2010). Bile duct ligation: step-by-step to cholangiocyte inflammatory tumorigenesis. *European Journal of Gastroenterology and Hepatology*.

[6] Borovikova L. V., Ivanova S., Zhang M., Yang H., Botchkina G. I., Watkins L. R., Wang H., Abumrad N., Eaton J. W., Tracey K. J. (2000). Vagus nerve stimulation attenuates the systemic inflammatory response to endotoxin. *Nature*.

[7] de Jonge W. J., Ulloa L. (2007). The alpha7 nicotinic acetylcholine receptor as a pharmacological target for inflammation. *British Journal of Pharmacology*.

[8] Marrero M. B., Bencherif M., Lippiello P. M., Lucas R. (2011). Application of alpha7 nicotinic acetylcholine receptor agonists in inflammatory diseases: an overview. *Pharmaceutical Research*.

[9] Liu C., Shen F.-M., Le Y.-Y., Kong Y., Liu X., Cai G.-J., Chen A. F., Su D.-F. (2009). Antishock effect of anisodamine involves a novel pathway for activating *α*7 nicotinic acetylcholine receptor. *Critical Care Medicine*.

[10] Hofer S., Eisenbach C., Lukic I. K., Schneider L., Bode K., Brueckmann M., Mautner S., Wente M. N., Encke J., Werner J., Dalpke A. H., Stremmel W., Nawroth P. P., Martin E., Krammer P. H., Bierhaus A., Weigand M. A. (2008). Pharmacologic cholinesterase inhibition improves survival in experimental sepsis. *Critical Care Medicine*.

[11] Liu A. J., Zang P., Guo J. M., Wang W., Dong W. Z., Guo W., Xiong Z. G., Wang W. Z., Su D. F. (2012). Involvement of acetylcholine-*α*7nAChR in the protective effects of arterial baroreflex against ischemic stroke. *CNS Neuroscience and Therapeutics*.

[12] Sun L., Zhang G.-F., Zhang X., Liu Q., Liu J.-G., Su D.-F., Liu C. (2012). Combined administration of anisodamine and neostigmine produces anti-shock effects: involvement of *α*7 nicotinic acetylcholine receptors. *Acta Pharmacologica Sinica*.

[13] Zhou J.-X., Ke P., Huan G., Shao B.-Z., Liu C. (2014). Combined treatment with anisodamine and neostigmine inhibits joint inflammation in collagen-induced arthritis mice. *CNS Neuroscience and Therapeutics*.

[14] Higgins G. M., Anderson R. M. (1931). Experimental pathology of liver resection. *Archives of Pathology*.

[15] de Graaf W., Heger M., Spruijt O., Maas A., de Bruin K., Hoekstra R., Bennink R. J., van Gulik T. M. (2012). Quantitative assessment of liver function after ischemia-reperfusion injury and partial hepatectomy in rats. *Journal of Surgical Research*.

[16] Livak K. J., Schmittgen T. D. (2001). Analysis of relative gene expression data using real-time quantitative PCR and the 2-ΔΔCT method. *Methods*.

[17] Poupko J. M., Baskin S. I., Moore E. (2007). The pharmacological properties of anisodamine. *Journal of Applied Toxicology*.

[18] Wang S.-T., Kuo N.-L. (1978). Experience in emergency treatment of shock due to infection. *Chinese Medical Journal*.

[19] Rui-Juan X. (1980). Studies on microcirculation in Institute of Basic Medical Sciences, Chinese Academy of Medical Sciences. *Microvascular Research*.

[20] Lalor P. F., Shields P. S., Grant A. J., Adams D. H. (2002). Recruitment of lymphocytes to the human liver. *Immunology and Cell Biology*.

[21] van Sweringen H. L., Sakai N., Tevar A. D., Burns J. M., Edwards M. J., Lentsch A. B. (2011). CXC chemokine signaling in the liver: impact on repair and regeneration. *Hepatology*.

[22] Tracy T. F., Bailey P. V., Goerke M. E., Sotelo-Avila C., Weber T. R. (1991). Cholestasis without cirrhosis alters regulatory liver gene expression and inhibits hepatic regeneration. *Surgery*.

[23] Dixon J. M., Armstrong C. P., Duffy S. W., Davies G. C. (1983). Factors affecting morbidity and mortality after surgery for obstructive jaundice: a review of 373 patients. *Gut*.

[24] Liu T.-Z., Lee K.-T., Chern C.-L., Cheng J.-T., Stern A., Tsai L.-Y. (2001). Free radical-triggered hepatic injury of experimental obstructive jaundice of rats involves overproduction of proinflammatory cytokines and enhanced activation of nuclear factor *κ*B. *Annals of Clinical and Laboratory Science*.

[25] Zhou W., Chao W., Levine B. A., Olson M. S. (1992). Role of platelet-activating factor in hepatic responses after bile duct ligation in rats. *The American Journal of Physiology—Gastrointestinal and Liver Physiology*.

[26] Levy R., Schlaeffer F., Keynan A., Nagauker O., Yaari A., Sikuler E. (1993). Increased neutrophil function induced by bile duct ligation in a rat model. *Hepatology*.

[27] Lechner A. J., Velasquez A., Knudsen K. R., Johanns C. A., Tracy T. F., Matuschak G. M. (1998). Cholestatic liver injury increases circulating TNF-*α* and IL-6 and mortality after *Escherichia coli* endotoxemia. *The American Journal of Respiratory and Critical Care Medicine*.

[28] DeLeve L. D. (2013). Liver sinusoidal endothelial cells and liver regeneration. *Journal of Clinical Investigation*.

[29] Stehr A., Ploner F., Traeger K., Theisen M., Zuelke C., Radermacher P., Matejovic M. (2005). Plasma disappearance of indocyanine green: a marker for excretory liver function?. *Intensive Care Medicine*.

[30] Mizuno S., Isaji S. (2010). Indocyanine green (ICG) fluorescence imaging-guided cholangiography for donor hepatectomy in living donor liver transplantation. *The American Journal of Transplantation*.

[31] Yoshidome H., Miyazaki M., Shimizu H., Ito H., Nakagawa K., Ambiru S., Nakajima N., Edwards M. J., Lentsch A. B. (2000). Obstructive jaundice impairs hepatic sinusoidal endothelial cell function and renders liver susceptible to hepatic ischemia/reperfusion. *Journal of Hepatology*.

[32] Akinci S. B., Ulu N., Yondem O. Z., Firat P., Guc M. O., Kanbak M., Aypar U. (2005). Effect of neostigmine on organ injury in murine endotoxemia: missing facts about the cholinergic antiinflammatory pathway. *World Journal of Surgery*.

